# Duplicated Inferior Vena Cava: A Case Study With Clinical Implications

**DOI:** 10.7759/cureus.81652

**Published:** 2025-04-03

**Authors:** Hamsat Anwar, Andrew Pardi, Mira Patel, Aaishah R Raquib, Katherine Andrews, Christine Broniak, Edith (Edie) Sperling

**Affiliations:** 1 Medicine, Western University of Health Sciences COMP-NW (College of Osteopathic Medicine of the Pacific – Northwest), Portland, USA; 2 College of Medicine, Western University of Health Sciences, Lebanon, USA; 3 Surgery, Desert Regional Medical Center, Palm Springs, USA; 4 Anatomical Sciences, Western University of Health Sciences, Lebanon, USA

**Keywords:** abdominal imaging, cadaver dissection, clinical and functional anatomy, double inferior vena cava, pulmonary emboli, venous thromboembolism

## Abstract

The inferior vena cava (IVC) is a large, retroperitoneal vein that ascends anterolaterally along the right side of the vertebral column, draining venous blood from both lower extremities, the pelvis, and the abdomen to the right atrium of the heart. The IVC undergoes complex embryological development during the fourth to eighth weeks of gestation, which can result in many anatomical variations, including duplication of the IVC. This study presents the case of duplicated IVCs in a 79-year-old male donor patient. While the second IVC on the left side of the body generally terminates by draining into the left renal vein, the individual described here demonstrated an extension superior to the renal veins, with the renal and gonadal veins draining into the ipsilateral IVCs. The two IVCs joined at T9 and passed as one into the right atrium at T8. The IVC’s role as the largest vein in the body is important to consider when assessing any vascular abnormality present in the IVC and its potential implications, which may include a higher risk of venous thromboembolism or pulmonary embolism.

## Introduction

The inferior vena cava (IVC) is a large retroperitoneal vein that ascends along the right side of the vertebral column. It is typically formed from a confluence of the left and right iliac veins and drains deoxygenated blood from the abdomen, pelvis, and lower extremities to the right atrium of the heart [1]. The IVC undergoes complex embryological development during the fourth to eighth weeks of gestation [2]. During this time, three pairs of embryonic veins arise in sequential order: the posterior cardinal veins, the subcardinal veins, and the supracardinal veins [2]. Anastomoses of these venous networks, as well as regression of select segments, occur to form the standard right-sided IVC [3,4]. The suprarenal portion of the IVC is formed by the suprarenal segment of the subcardinal vein, the renal portion is formed by the fusion of the renal segments from the bilateral subcardinal veins, and the infrarenal portion is formed by the right supracardinal vein [3].

Normally, the left supracardinal vein regresses inferiorly to the renal segment [2,4]. Ultimately, the IVC, located on the posterior abdominal wall, is created by the joining of the common iliac veins and runs adjacent to the right side of the abdominal aorta [1]. While traversing the abdominal cavity and entering the thoracic cavity, it collects venous return from the inferior phrenic, right suprarenal, renal, right gonadal, lumbar, common iliac, and hepatic veins [1]. Anatomical variation arises when certain subcardinal or supracardinal veins irregularly persist or regress [2]. A duplicated IVC, as seen in the individual presented here, occurs due to abnormal persistence of both the right and left supracardinal veins [2]. In a duplicated IVC, the left IVC usually ends by joining the left renal vein, which then crosses the aorta and enters the typical right-sided IVC [4-6]. Other variations include a continuation of the right-sided IVC into the azygos vein, which drains the thoracic cage into the superior vena cava, or the complete absence of an IVC [1,5].

This study presents the case of a duplicated IVC in a donor patient (cadaver), discussing the clinical significance, treatment guidelines, implications for health, and considerations related to vascular surgery. Given the rarity of any IVC variation (0.6-2%), there is limited research available to adequately address the presence of a virtually full-length duplicated IVC, with primarily case reports available [3]. Our goal was to clearly define the characteristics of this marked variation, compare it to the literature, and provide clinical insight into potential complications.

## Case presentation

**Figure 1:**
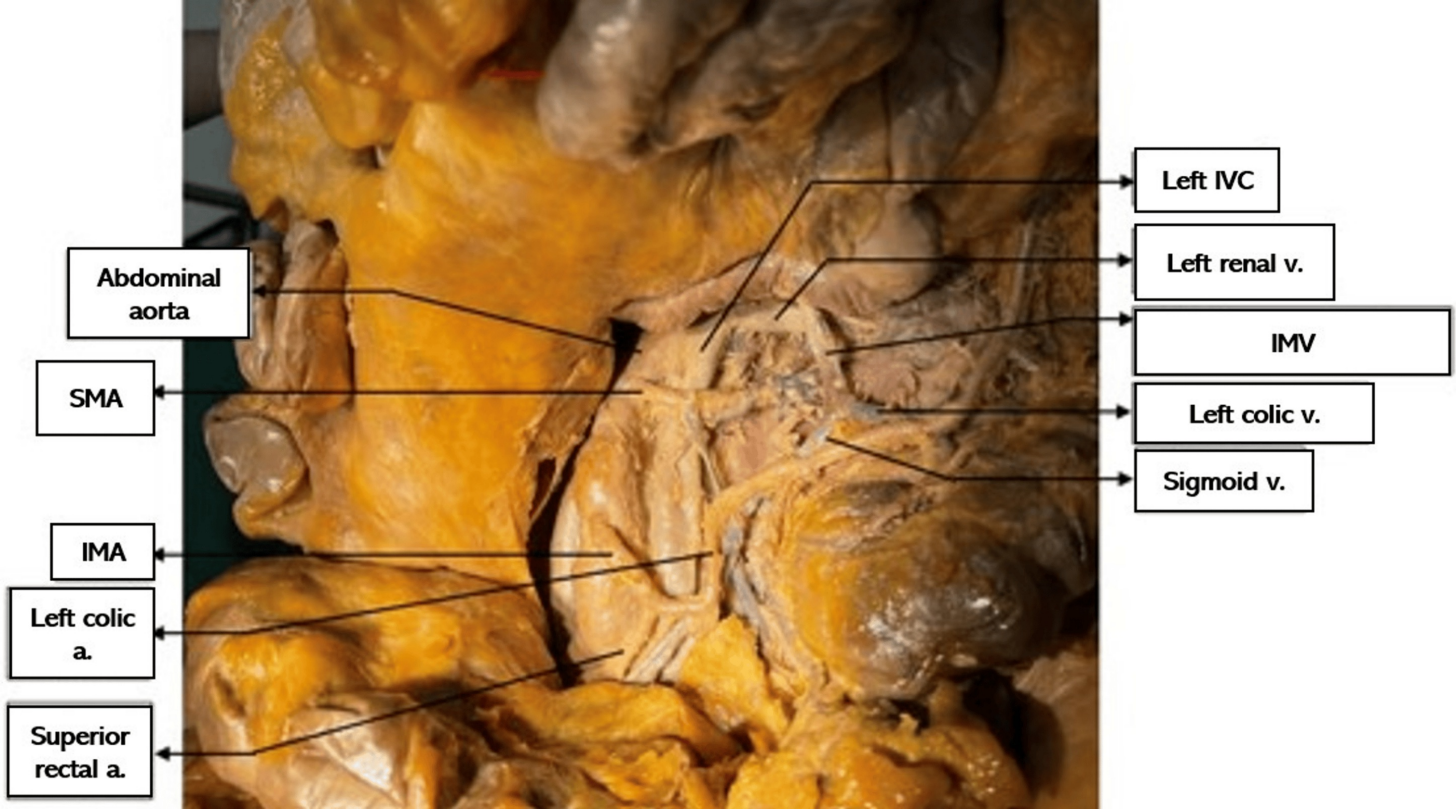
Left IVC a.: artery; IMA: inferior mesenteric artery; IVC: inferior vena cava; SMA: superior mesenteric artery; v.: vein

**Figure 2:**
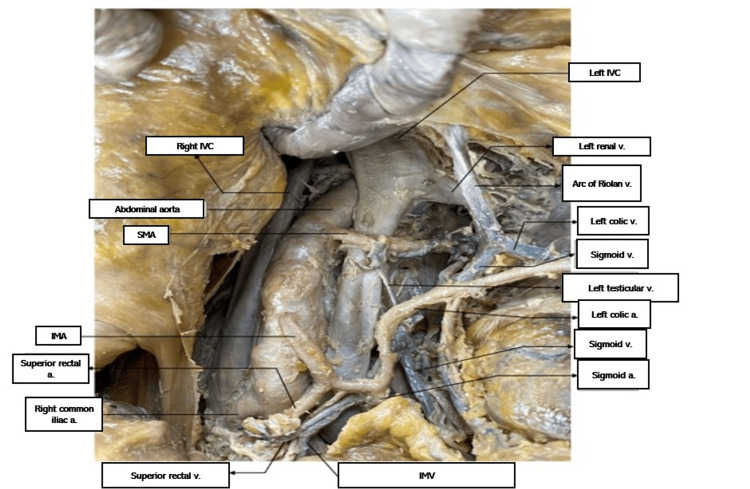
Duplicated IVCs a.: artery; IMA: inferior mesenteric artery; IMV: inferior mesenteric vein; IVC: inferior vena cava; SMA: superior mesenteric artery; v.: vein

**Figure 3:**
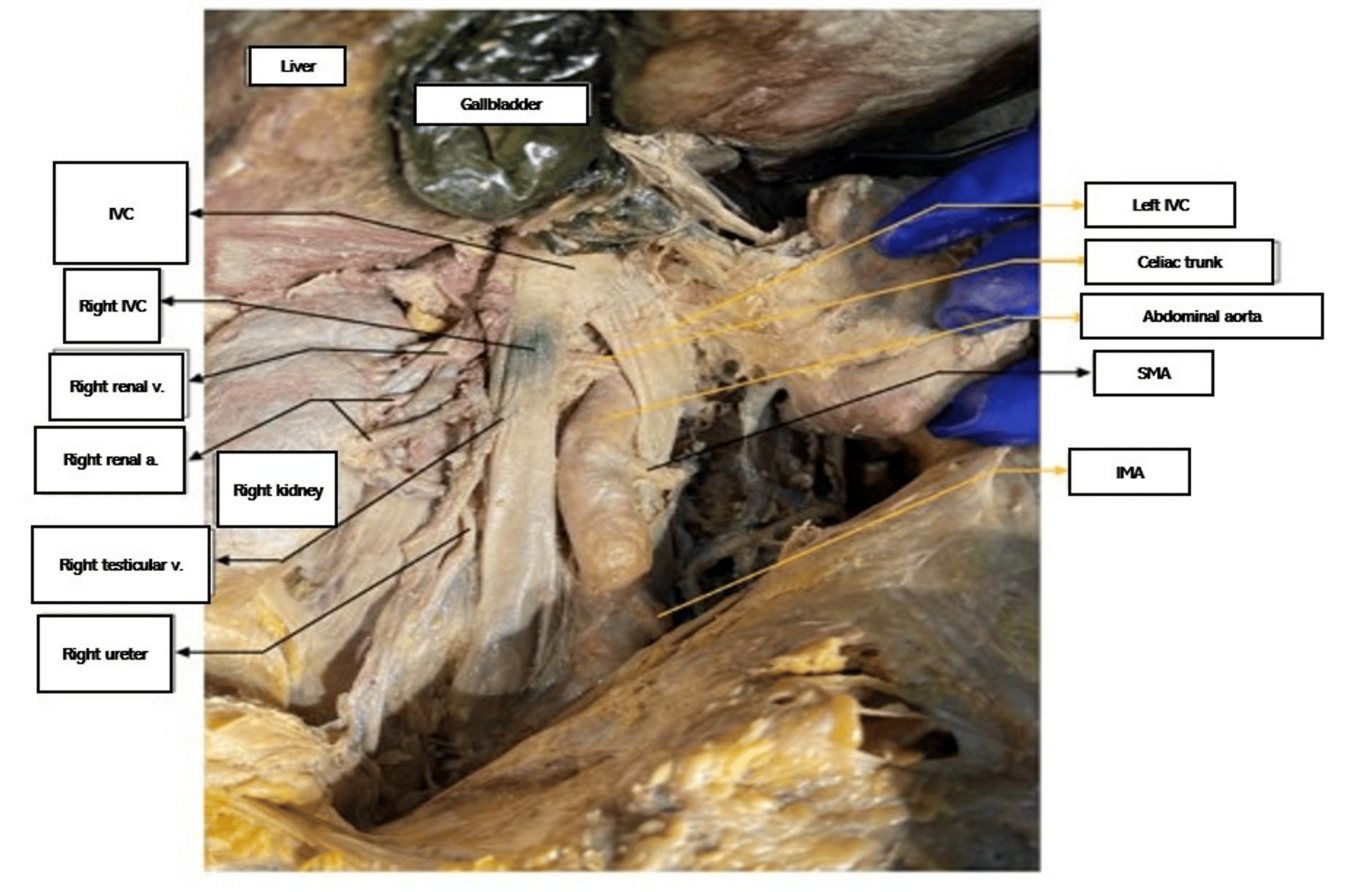
Joining of the two IVCs a.: artery; IMA: inferior mesenteric artery; IVC: inferior vena cava; SMA: superior mesenteric a.; v.: vein

This study was approved as non-human subject research by the Institutional Review Board of the Western University of Health Sciences (#1924683). After death, a 79-year-old male donor patient was taken per his wishes to the Willed Body program at Western University of Health Sciences. He was embalmed with a formalin and water mixture. Upon routine dissection by medical students, the donor patient was found to have two equal-sized IVCs on the right and left sides of the spinal column anterolaterally, each vessel arising separately and directly as continuations of the right and left common iliac veins. The left IVC crossed the abdominal aorta at T10, and the IVCs joined at T9 to enter the right atrium as usual at T8. See Figures 1-3 for visuals of the donor.

The left renal vein and left gonadal vein drained into the left IVC. The right renal and right gonadal vein drained into the right IVC as usual. The celiac trunk arose from the abdominal aorta between the two IVCs just distal to the joining of the IVCs, and the splenic artery passed under the left IVC.

## Discussion

While the vast majority of IVC congenital abnormalities are asymptomatic and pose no clinical complications, there are reports of duplication of the IVC associated with venous thromboembolism (VTE) [7,8]. The correlation and etiology of this specific duplication of the IVC with VTE is currently unknown, but case reports suggest individuals with duplicated IVCs are predisposed to venous stasis and chronic venous insufficiency [7,8]. Duplication of the IVC alone is not sufficient to compose a primary risk factor for VTE, but coupled with other risk factors, such as circulatory insufficiency, factor V Leiden, or prolonged immobility, the risk of VTE may be significantly higher [7]. This is highlighted by the observed trend of individuals with duplication of the IVC experiencing their first thrombosis at age 35 or younger [7].

In addition to this association, CT or ultrasound of patients who present with acute symptoms of venous thromboembolism is greatly complicated by the presence of an undiscovered duplication of the IVC [7]. The appearance of this vascular abnormality has been confused with retroperitoneal cysts, aorto-lumbar lymphadenopathy, aortic aneurysm, and dilation of the left ureter, leading to misdiagnosis and critical delay in treatment in some cases [7]. The accepted “gold standard” for diagnosis, therefore, remains phlebography with contrast over combination CT and ultrasound; however, the invasive nature of this technique may not be appropriate in all situations and, therefore, limits its use [9].

In the presence of VTE or pulmonary embolism (PE), duplication of the IVC provides not only a chance for misdiagnosis but potential adjustment to the treatment plan as well [7]. While a typical patient with lower extremity VTE may present with the classic signs of lower extremity edema and claudication, individuals with a duplicated IVC may sometimes report initial symptoms associated with the thorax, abdomen, and genital region [7]. In patients with recurrent PE and VTE unresponsive to anti-coagulant therapy, the placement of a retrievable wire filter within the IVC to catch thrombi dislodged from the lower extremity has become increasingly common over the past two decades [10]. This device is placed endovascularly, with catheterization of the femoral vein to obtain venous access [10]. In patients with duplicated IVCs, standard placement will only filter blood flow from one of the lower extremities, allowing free flow of any clots rising from the patient’s other side [10].

Further case studies have reported on the presence of a double IVC within the context pathologies, including reninoma [11], renal cell carcinoma [12,13], gastric tumor [14], and ureteral obstruction [15]. The diagnosis of a reninoma may involve renal vein sampling, which is made much more difficult by the presence of two IVCs and may affect the results [11]. Extension of renal cell carcinoma into both IVCs has also been reported, which in one case necessitated removal of the left IVC [12] and in the other case, vena caval thrombectomy [13]. In the case of the ureteral obstruction, pre-surgical radiographs appeared to show a retrocaval ureter, but upon surgical exploration, an incomplete duplication of the IVC, which was causing the obstruction, was found [15]. Surgical release of the vessel was performed successfully, and the authors recommend keeping a duplicated IVC in the diagnostic differential for extrinsic ureteral obstruction [15]. Spiral CT and three-dimensional reconstruction of spiral CT may assist with clearly delineating the anatomy of retroperitoneal variations prior to surgery [13,16].

Failure to diagnose duplication is exacerbated by the lack of surgical guidelines related to addressing duplicated IVC and may lead to treatment failure, continued recurrence of PEs, and in some cases, death [9,10,17]. While rare, these issues can be avoided by accurate phlebography and addressed by the placement of filtration devices bilaterally or, in some cases, ligation and embolization of the left IVC [9]. While further data are needed to assess links between early detection and improved surgical outcomes, we suggest that patients under the age of 35 who are at high risk for thrombosis, such as those with factor V Leiden and prothrombin gene mutation, could be potential candidates for asymptomatic diagnostic imaging. While duplication of the IVC is rare, compounding risk factors are quickly exacerbated by unclear changes to hemodynamics and the chance for critical errors, both diagnostically and operatively.

## Conclusions

This case report describes the finding of a duplicated IVC in a 79-year-old male donor patient during routine dissection in an anatomy lab, in whom the two IVCs were continuations of the right and left common iliac veins, joining together at T9 before passing as one IVC into the right ventricle at T8. We discuss the implications of a duplicated IVC, including incidents of VTE and PE, in which the duplicated IVC should be known prior to intervention to guide the treatment plan and minimize risk. The lack of both diagnostic and treatment guidelines indicates that the link between venous stasis and duplication of the IVC would benefit from further research.
